# Supplementary material for: Adapting to time: Why nature may have evolved a diverse set of neurons
Source: PLoS Comput Biol. 2024 Dec 13;20(12):e1012673. doi: 10.1371/journal.pcbi.1012673 (PMC11676785; doi:10.1371/journal.pcbi.1012673)
Supplement: S1 Text — (PDF) [file pcbi.1012673.s001.pdf]

Adapting to time: why nature may have evolved a diverse set of neurons

Supplementary material

December 3, 2024

List of Figures

1 Bursting example 8

List of Tables

1 Data for Fig 2A and 2B 3
2 Data for Fig 3A 4
3 Data for Fig 3B 4
4 Data for Fig 3D 5
5 Data for Fig 4A 5
6 Data for Fig 4B 6
7 Data for Fig 5A 6
8 Data for Fig 5B 7
9 Data for Fig 6A and 6B 7
10 Data for Fig 6C 8

General note, for all simulations of this work we used four 3090 GPUs with elite sharing. Specifically, The top elites/4 from each GPU are shared between all GPUs after each generation to seed better solutions.

Section 1 Fig. 2 supplementary material

For Figs 2A and 2B in the main text, all simulations used an architecture of (2, 4, 1) == (input, hidden, output). The population size is 1,840,000 solutions (460,000 * 4 GPUs). The elites are 4000 per GPU.

In Table A, we show more details about the simulations. Where, the afterpotential is fixed at 0 mV. The weights clipping is part of the simulations and not listed below. When not mutated, the weights are fixed at 1 mV, the time constants at 5 ms and the change in delays at 0 ms.

Experiment	Net-Arch	No. para	W-rate	$\mathcal{T}_c$-rate	$\mathcal{T}_c$-clip	D-rate	D-clip
W	(2, 4, 1)	12	0.25	2	(0.1, 10)	1	(-4, 4)
$W\mathcal{T}_C$	(2, 4, 1)	24	0.25	2	(0.1, 10)	1	(-4, 4)
$D\mathcal{T}_C$	(2, 4, 1)	24	0.25	2	(0.1, 10)	1	(-4, 4)
WD	(2, 4, 1)	24	0.25	2	(0.1, 10)	1	(-4, 4)
$WD\mathcal{T}_C$	(2, 4, 1)	36	0.25	2	(0.1, 10)	1	(-4, 4)

Table A. Data for Figs 2A and 2B

In this table, parameter-rate is the mutation rate and parameter-clip is the parameter clipping range after mutation. Fig 2C is one of the solutions from Fig 2A $W\mathcal{T}_c$ plot.

Section 2 Fig. 3 supplementary material

For Fig 3A in the main text, all simulations used an architecture of (2, 6, 1) == (input, hidden, output). The population size is 1,120,000 solutions (280,000 * 4 GPUs). The elites are 2400 per GPU.

In Table B, we show more details about the simulations. Where, the afterpotential is fixed at 0 mV. The weights clipping is (-1, 1) mV. When not mutated, the weights are fixed at 1 mV, the time constants at 2 ms and the change in delays at 0 ms.

Experiment	Net-Arch	No. para	W-rate	$\mathcal{T}_c$-rate	$\mathcal{T}_c$-clip	D-rate	D-clip
W	(2, 6, 1)	18	0.25	4	(0.1, 10)	3	(-4, 4)
$W\mathcal{T}_C$	(2, 6, 1)	36	0.25	4	(0.1, 10)	3	(-4, 4)
$D\mathcal{T}_C$	(2, 6, 1)	36	0.25	4	(0.1, 10)	3	(-4, 4)
WD	(2, 6, 1)	36	0.25	4	(0.1, 10)	3	(-4, 4)
$WD\mathcal{T}_C$	(2, 6, 1)	54	0.25	4	(0.1, 10)	3	(-4, 4)

Table B. Data for Fig 3A

For Fig 3B in the main text, all simulations used an architecture of (2, 4, 1) == (input, hidden, output). The population size is 1,520,000 solutions (380,000 * 4 GPUs). The elites are 4000 per GPU.

In Table C, we show more details about the simulations. Where, the afterpotential is fixed at 0 mV. We only mutate the weights and change in delays, with the time constants held at 2 ms. The input encoding is (001, 011)==(No, Yes). The output is the number of spikes and its encoding is (1, 2)==(No, Yes).

Experiment	Net-Arch	No. para	W-rate	$\mathcal{T}_c$-rate	$\mathcal{T}_c$-clip	D-rate	D-clip
$\pm$0.8 mV	(2, 4, 1)	24	0.25	4	(0.1, 10)	3	(-4, 4)
$\pm$0.9 mV	(2, 4, 1)	24	0.25	4	(0.1, 10)	3	(-4, 4)
$\pm$1.2 mV	(2, 4, 1)	24	0.25	4	(0.1, 10)	3	(-4, 4)
$\pm$1.3 mV	(2, 4, 1)	24	0.25	4	(0.1, 10)	3	(-4, 4)

Table C. Data for Fig 3B

For Fig 3D in the main text, all simulations used an architecture of (2, 6, 1) == (input, hidden,

output). The population size is 1,024,000 solutions (256,000 * 4 GPUs). The elites are 4000 per GPU.

In Table D, we show more details about the simulations. Where, the afterpotential is fixed at 0 mV. The weights clipping is (-1, 1) mV. When not mutated, the weights are fixed at 1 mV and the time constants at 2 ms. The input encoding is (011, 111)==(No, Yes).

Experiment	Net-Arch	No. para	W-rate	$\mathcal{T}_c$-rate	$\mathcal{T}_c$-clip	D-rate	D-clip
WD	(2, 6, 1)	36	0.25	4	(0.1, 10)	3	(-4, 4)
$\mathcal{T}_C D$	(2, 6, 1)	36	0.25	4	(0.1, 10)	3	(-4, 4)

Table D. Data for Fig 3D

Section 3 Fig. 4 supplementary material

For Fig 4A in the main text, all simulations used an architecture of (2, 4, 1) == (input, hidden, output). The population size is 640,000 solutions (160,000 * 4 GPUs). The elites are 2000 per GPU.

In Table E, we show more details about the simulations. Where, the afterpotential is fixed at 0 mV. We only mutate the weights and change in delays. The output is the number of spikes and its encoding is (1, 2)==(No, Yes). We only use one logic gate and that is the 'XOR'.

Experiment	Net-Arch	No. para	W-rate	$\mathcal{T}_c$-rate	$\mathcal{T}_c$-clip	D-rate	D-clip
±0.6 mV	(2, 4, 1)	24	0.25	3	(0.1, 10)	2	(-4, 4)
±0.7 mV	(2, 4, 1)	24	0.25	3	(0.1, 10)	2	(-4, 4)
±0.8 mV	(2, 4, 1)	24	0.25	3	(0.1, 10)	2	(-4, 4)
±0.9 mV	(2, 4, 1)	24	0.25	3	(0.1, 10)	2	(-4, 4)

Table E. Data for Fig 4A

For Fig 4B in the main text, all simulations used an architecture of (2, 4, 1) == (input, hidden, output). The population size is 640,000 solutions (160,000 * 4 GPUs). The elites are 2000 per GPU.

In Table F, we show more details about the simulations. Where, the afterpotential is fixed at 0 mV. We only mutate the weights and change in delays.The input encoding is (001, 101)==(No, Yes). The output is the number of spikes and its encoding is (1, 2)==(No, Yes).

Experiment	Net-Arch	No. para	W-rate	$\mathcal{T}_c$-rate	$\mathcal{T}_c$-clip	D-rate	D-clip
±0.7 mV	(2, 4, 1)	24	0.25	3	(0.1, 10)	2	(-4, 4)
±0.8 mV	(2, 4, 1)	24	0.25	3	(0.1, 10)	2	(-4, 4)
±0.9 mV	(2, 4, 1)	24	0.25	3	(0.1, 10)	2	(-4, 4)

Table F. Data for Fig 4B

Section 4 Fig. 5 supplementary material

For Fig 5A in the main text, all simulations used an architecture of (2, 4, 1) == (input, hidden, output). The population size is 720,000 solutions (180,000 * 4 GPUs). The elites are 3000 per GPU. Each cell in the grid (loss grid in the figure) is the average of 32 trials (32 Epochs).

In Table G, we show more details about the simulations. Where, the afterpotential is fixed at 0 mV. The input encoding is (01111, 11111)==(No, Yes). The output is the number of spikes and its encoding is (1, 2)==(No, Yes). Large number of spikes in the input is needed to observe noise effects. However, it should be noted that, the longer the spike train, the smaller is the population size one can fit in a GPU. We only use one logic gate and that is the 'XOR'. The weights clipping is (-1, 1) mV.

Experiment	Net-Arch	No. para	W-rate	$\mathcal{T}_c$-rate	$\mathcal{T}_c$-clip	D-rate	D-clip
WD	(2, 4, 1)	24	0.25	3	(0.1, 10)	2	(-5, 5)
$W\mathcal{T}_C$	(2, 4, 1)	24	0.25	3	(0.1, 10)	2	(-5, 5)
$D\mathcal{T}_C$	(2, 4, 1)	24	0.25	3	(0.1, 10)	2	(-5, 5)
$WD\mathcal{T}_C$	(2, 4, 1)	36	0.25	3	(0.1, 10)	2	(-5, 5)

Table G. Data for Fig 5A

For Fig 5B in the main text, all simulations used an architecture of (2, 4, 1) == (input, hidden, output). The population size is 7,200 solutions (1,800 * 4 GPUs). The elites are 100 per GPU. Each cell in the grid (loss grid in the figure) is the average of 100 trials.

In Table H, we show more details about the simulations. Where, the afterpotential is fixed at 0 mV. The input encoding is (01111, 11111)==(No, Yes). The output is the number of spikes and its encoding is (1, 2)==(No, Yes). We only use one logic gate and that is the 'XOR'. The weights clipping is (-1, 1) mV.

Experiment	Net-Arch	No. para	W-rate	$\mathcal{T}_c$-rate	$\mathcal{T}_c$-clip	D-rate	D-clip
W	(2, 4, 1)	24	0.25	3	(0.1, 10)	2	(-5, 5)
WD	(2, 4, 1)	24	0.25	3	(0.1, 10)	2	(-5, 5)
$W\mathcal{T}_C$	(2, 4, 1)	24	0.25	3	(0.1, 10)	2	(-5, 5)
$WD\mathcal{T}_C$	(2, 4, 1)	36	0.25	3	(0.1, 10)	2	(-5, 5)

Table H. Data for Fig 5B

Section 5 Fig. 6 supplementary material

For Fig 6A and 6B in the main text, all simulations used an architecture of (2, 6, 1) == (input, hidden, output). The population size is 1,080,000 solutions (270,000 * 4 GPUs). The elites are 3000 per GPU.

In Table I, we show more details about the simulations. The weights clipping is (-1, 1) mV. When not mutated, the weights are fixed at 1 mV, the time constants at 5 ms ,the change in delays at 0 ms and the afterpotential at 0 mV.

Experiment	Net-Arch	No. para	W-rate	$\mathcal{T}_c$-rate	$\mathcal{T}_c$-clip	D-rate	D-clip
$W\mathcal{T}_C$	(2, 6, 1)	36	0.25	2	(0.1, 10)	1	(-4, 4)
$D\mathcal{T}_C$	(2, 6, 1)	36	0.25	2	(0.1, 10)	1	(-4, 4)
WD	(2, 6, 1)	36	0.25	2	(0.1, 10)	1	(-4, 4)
$WD\mathcal{T}_C$	(2, 6, 1)	54	0.25	2	(0.1, 10)	1	(-4, 4)
$W\beta D\mathcal{T}_C$	(2, 6, 1)	72	0.25	2	(0.1, 10)	1	(-4, 4)

Table I. Data for Fig 6A and 6B

In Fig A, we show how the spiking afterpotential can motivate a neuron to fire repeatedly after a threshold crossing as seen from neurons three, five and nine. In this example, the target is a spike train and all parameters are adapted, namely; weights, afterpotential, time constants and delays. This example is a sample solution from the very most right column of Fig 6B in the main text ($W\beta D\mathcal{T}_C$ case).

A)

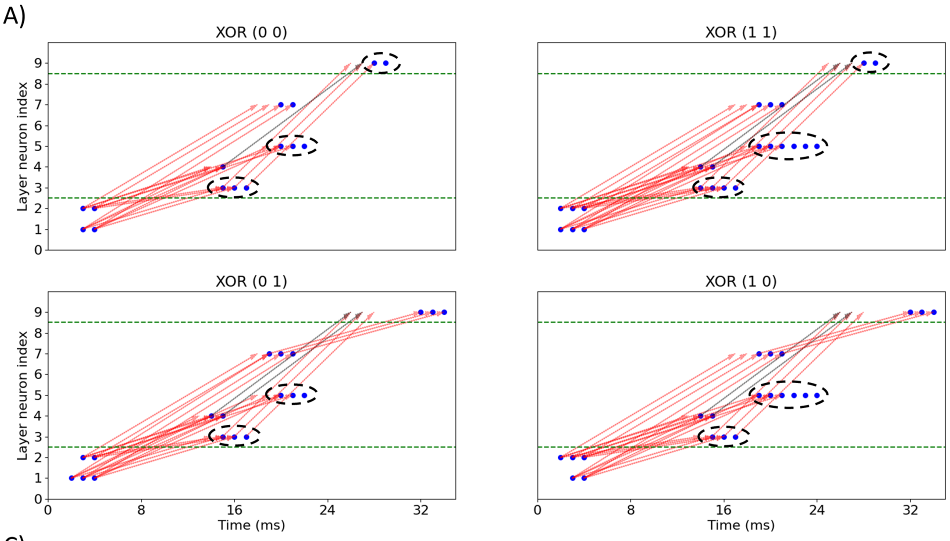

B)

Layer 2-W	
3	0.96
4	-1.00
5	-0.53
6	0.82
7	0.69
8	0.44

Layer 2-TC	
3	9.60
4	5.50
5	3.58
6	3.07
7	10.00
8	2.56

Layer 2-B	
3	-0.16
4	-0.05
5	-0.06
6	0.00
7	-0.27
8	0.00

Layer 2-D	
3	-4.00
4	-2.82
5	3.01
6	3.68
7	-1.69
8	4.00

C)

Input	Layer 1-W					
1	1.00	0.15	0.26	-1.00	0.92	-0.64
2	0.74	0.88	0.87	-0.65	0.46	0.08
Output	3	4	5	6	7	8

Input	Layer 1-TC					
1	5.66	7.88	0.10	0.10	5.03	7.79
2	7.08	3.77	10.00	6.50	8.18	2.12
Output	3	4	5	6	7	8

Input	Layer 1-B					
1	-0.01	-0.13	-0.07	-0.40	-0.14	-0.12
2	-0.37	-0.28	-0.03	0.00	-0.32	-0.10
Output	3	4	5	6	7	8

Input	Layer 1-D					
1	-3.08	-3.74	1.42	-4.00	1.72	-3.38
2	-2.95	-3.78	2.22	1.10	0.21	3.23
Output	3	4	5	6	7	8

Fig A. A spiking raster plot showing the bursting behavior. A) the spiking plots for all input/output combinations for an `XOR` example. B) the second layer and C) the first layer weights, afterpotentials, time constants and delays. Abbreviations code, W: weights, B: spiking afterpotential, TC: time constants and D: delays.

For Fig 6C in the main text, all simulations used an architecture of (2, 4, 1) == (input, hidden, output). The population size is 512,000 solutions (128,000 * 4 GPUs). The elites are 2000 per GPU.

In Table J, we show more details about the simulations. The weights clipping is (-1, 1) mV. We mutate the weights, time constants and delays, with the afterpotential at 0 mV. The input encoding is (001, 011)==(No, Yes). The time constant for the decay of the output objective is 5 ms.

Experiment	Net-Arch	No. para	W-rate	$\mathcal{T}_c$-rate	$\mathcal{T}_c$-clip	D-rate	D-clip
$WD\mathcal{T}_C$	(2, 4, 1)	36	0.25	4	(0.1, 10)	4	(-10, 10)

Table J. Data for Fig 6C
