# Supplementary material for: Adapting to time: Why nature may have evolved a diverse set of neurons
Source: PLoS Comput Biol. 2024 Dec 13;20(12):e1012673. doi: 10.1371/journal.pcbi.1012673 (PMC11676785; doi:10.1371/journal.pcbi.1012673)
Supplement: Attachment — Submitted filename: PCB_time_constant_review.pdf [file pcbi.1012673.s002.pdf]

# 1 Overall impression

Are the temporal processing features of biological neural networks computationally useful, even for tasks that lack an intrinsic temporal component? To answer this important question, Habashy et al. compared how easily spiking neural networks could be trained to perform basic logical operations (AND, OR, XOR, etc.) by adjusting only the weights of connections between neurons (referred to as spatial parameters), only the membrane time constants and axonal conduction delays (referred to as temporal parameters), or both. One of their most thought-provoking results, as highlighted in the abstract, is that it was possible to successfully train networks to solve all tested tasks by changing only temporal parameters while holding the weights fixed. This result on its own will spark many interesting discussions, including among non-computational neuroscientists. The authors go on to explore sources of degeneracy in their model, the impact of varying certain hyperparameters, and robustness to different types of noise; a thorough set of computational experiments that are relevant to a more specialized audience. I enjoyed this paper from the first read, and I believe others will enjoy it as well.

# 2 Scientific criticisms

The title and conclusion of the abstract suggest that this work offers an explanation of how or why diverse membrane time constants and axonal conduction delays evolved in the brain. In my opinion, substantiating such an explanation using a computational approach would require a clear model of the evolutionary processes and pressures acting on biological neural systems and/or a clear example of a function that can be performed by a neural network with diverse temporal processing features but not by a comparable network without those features. Unfortunately, I am not yet convinced that this manuscript offers either.

- **Essential revision:** The only connection between this work and the evolution of biological neural networks seems to be the use of an evolutionary algorithm for optimization. This strikes me as a fairly superficial connection, perhaps because I am not an expert in such things. I would have a better appreciation for the significance of choosing an evolutionary algorithm if the authors included a discussion of the specific aspects of biological evolution that the tool they use is intended to mimic, and/or if the authors could point to any aspects of their work that are not achievable using synthetic gradient descent (https://zenodo.org/badge/latestdoi/170391179).

- **Essential revision:** Fig. 2A seems to show that certain logical operations can be learned by training temporal parameters (middle column) but not by training weights (left column). I find this a very surprising result, partly because spiking neural networks have previously been trained to solve much more complex tasks using only weights (Neftci et al., *IEEE Signal Processing Magazine*, 2019; Cramer et al., *IEEE Transactions on Neural Networks and Learning Systems*, 2022; Harkin et al., *Neuroscience*, 2022). It would be helpful if the authors could provide a discussion of potential reasons for this negative result. Separately, the colorbar accompanying this figure seems to indicate that many of the solutions were achieved in generation zero (darkest shade of blue), making me worry that the apparent differences between conditions could be due to (un)lucky initialization rather than training. It would be helpful if the authors could provide clarification on this point.

- **Non-essential revision:** Neural networks are so flexible (Cybenko, *Mathematics of Control, Signals and Systems*, 1989; Siegelmann et al., *Proceedings of the fifth annual workshop on Computational learning theory*, 1992; Kawaguchi et al., *Neural Computation*, 2019) that I am personally sceptical of strong claims that "network $X$ cannot do $Y$, but network $Z$ can" on the basis of purely empirical results, as in your line 150. This is a subjective point, but I would prefer if such conclusions were worded in a way that reflects the empirical rather than theoretical nature of this work ("we did not observe any instances of $X$ doing $Y$").

If the above criticisms cannot be addressed, then this work is still interesting and important, but it does not explain how or why diverse time constants and axonal delays evolved in the brain. I would suggest

modifying the title and abstract to better align with the final two paragraphs of the discussion, which I feel better reflect the main themes of this work.

# 3 Presentation

While the manuscript is clearly written, the overall style feels slightly closer to that of a perspective article or review than to a research article. The methods and results are described at a fairly high level; as a result, I would not be able to reproduce this work without access to the code (which seems to be private at the time of writing), and I would find it difficult to point to any specific measurements that support the main conclusions. In particular, I could not find the following details:

- Basic aspects of network architecture including depth, width, and number of trainable parameters per condition.
- Definitions of $\delta, K, \alpha, \lambda$ in equations 1 and 2. All of these are guessable (except the value of $K$, which seems to be an important architectural parameter that represents the number of dendrites per neuron), but I think it would be better to explicitly define.
- Definition of the LIF reset rule. Again, known by specialists, but worth writing down.
- Evolutionary algorithm parameters, including exact initial population size, number of elites, and mutation rate.
- Time constant of the exponential decay kernel used for the spike train objective (line 128).
- Justification or citation for the low voltage threshold (1.1 mV) and assumption that dendrites have a longer time constant than somata (off the top of my head, I would have guessed the opposite due to lower dendritic capacitance).
- Meaning of the $y$-axis, arrows, and green lines in fig. 2C left, 3B right, and 3C right. I am not at all sure how to interpret these plots.
- Any type of uncertainty or error on any result. This is not as important here as it would be for a paper involving biological experiments, but the absence is conspicuous.

All of the above points are essential revisions, but should mostly not take much time.

# 4 Potential discussion points

The fact that even tasks without an intrinsic temporal structure must be solved over time is one of the most basic and often-ignored distinctions between biological and artificial neural networks, as rightly emphasized by Habashy et al. At the same time, there are some classes of artificial neural networks that deal explicitly with sequences (eg, liquid time constant networks, gated recurrent units, long short-term memory networks, and recurrent networks more generally). Including a brief discussion of potential similarities and differences between a feed-forward network with variable time constants and delays and a recurrent network architecture, and/or the potential impact of incorporating conduction delays into recurrent networks might make the manuscript more well-rounded. This is not an essential point, however.
