# Supplementary material for: Adapting to time: Why nature may have evolved a diverse set of neurons
Source: PLoS Comput Biol. 2024 Dec 13;20(12):e1012673. doi: 10.1371/journal.pcbi.1012673 (PMC11676785; doi:10.1371/journal.pcbi.1012673)
Supplement: Attachment — Submitted filename: Response Letter .pdf [file pcbi.1012673.s003.pdf]

Acknowledgments:

We would like to thank the reviewers and editors for their thoughtful comments on our paper originally entitled "Adapting to time: why nature evolved a diverse set of neurons". We very much appreciate the positive responses. We address the two reviewers in turn below and provide a "Diff file" to highlight the revisions in the manuscript.

Reviewer 1 comments with replies under:

1. **Reviewer 1 notes that both the title and conclusion of the abstract suggest that our work explains how or why diverse membrane time constants and axonal conduction delays evolved in the brain. He or she is not convinced that our findings substantiate these conclusions.**

   Reply: we agree we have not given any insight into *how* this diversity developed. However, we do think we have provided a potential explanation as to why diversity may have developed – namely, we show that adapting temporal parameters makes it easier to find solutions to a set of simple spatial-temporal tasks. We do not suggest that the specific tasks we have used drove this diversity. Nor do we claim that the specific evolutionary algorithm we used is a good match the processes that drove diversity in biological organisms. However, our broader claim – that adaptation of temporal parameters can make it easier to identify solutions to spatial-temporal problems – is supported by our findings. We have provided a proof of principle that fundamental computations can be learnt by changing properties other than synaptic weights (challenging the central dogma of learning and memory).

   We do take the point that our title and abstract can be taken as making stronger claims than we intend. Accordingly, we have modified our title to "Adapting to time: why nature **may have** evolved a diverse set of neurons". We have also highlighted more clearly the scope of our conclusions, not only on the abstract, but also in the discussion (see lines 420 on).

2. **Related to these concerns, the Reviewer requests two essential revisions. First, he or she is not convinced that there is anything special about the evolutionary algorithm that we used, and that similar results might be obtained if we had used gradient descent.**

   Reply: we agree that similar outcomes would likely be obtained. But again, our point is more general – modifying temporal parameters is useful when solving fundamental boolean logic gates in a spatial-temporal context, and we expect that this is true whichever algorithm is used. We chose a standard evolutionary algorithm for two reasons: a) evolutionary algorithms are easier to use in this context given that no gradients need to be computed, and b) the diversity of neurons in the brain is the product of evolution, so it seemed more elegant and fitting to use an (arbitrary) form of evolution to make our point. Again, we clarify our claims in our revision (see lines from 148 on).

3. **Second, the reviewer is surprised that adapting weights did not solve all our problems. He/she notes that more complex tasks have been solved by spiking networks when only adapting weights.**

   Reply: again, we agree, and we do not mean to suggest that there are no weight-based solutions to our tasks. In our simulations, we used tiny feed-forward networks (composed of 7-9 units), and under these conditions, perhaps temporal parameters are needed. We do not want to rule out the possibility that solutions would be found in our models if we evolved them for more generations, or if we used a different evolutionary or learning algorithm. The important point, however, is that adapting temporal parameters can make it much easier to evolve solutions. We take this as evidence that evolution may have exploited temporal parameters, even if purely spatial solutions are possible. Again, we make this point explicit in the revision (see lines from 185 on)

4. **Reviewer 1 also suggests a non-essential revision, namely, that we should not make absolute claims based on our empirical findings, noting a too strong claim of ours on line 150.**

   Reply: we agree and have amended our claims accordingly.

5. **Reviewer 1 also makes some points regarding the presentation. We first quote reviewer and then the response.**

   a) **"Basic aspects of network architecture including depth, width, and number of trainable parameters per condition".**

      Reply: this information has been added to the supplementary materials.

   b) **"Definitions of δ, K, α, λ in equations 1 and 2. All of these are guessable (except the value of K, which seems to be an important architectural parameter that represents the number of dendrites per neuron), but I think it would be better to explicitly define".**

      Reply: Information added on line 118. For α and λ, they are changed in the equations.

   c) **"Definition of the LIF reset rule. Again, known by specialists, but worth writing down".**

      Reply: Added on line 122.

   d) **"Evolutionary algorithm parameters, including exact initial population size, number of elites, and mutation rate".**

      Reply: Added in the supplementary materials.

e) **"Time constant of the exponential decay kernel used for the spike train objective (line 128)"**

   Reply: Added on line 137

f) **"Justification or citation for the low voltage threshold (1.1 mV) and assumption that dendrites have a longer time constant than somatic (off the top of my head, I would have guessed the opposite due to lower dendritic capacitance)".**

   Reply: regarding the threshold, our choice was arbitrary. The critical point was that across conditions we clipped weights above or below this threshold to assess the impact on the various temporal parameters. Regarding the time constants, the reviewer may well be correct for a small patch of dendrite, however it is different when considering low pass filtering induced by the whole associated axon-dendritic structure (Segev et al., 1994 section 4.2). This point is made clearer in the paper. In addition, we would note that: i) there are other studies that used this formalism (Tapson et al. 2016 and Beniaguev et al. 2022); ii) Synaptic/dendritic time constants were necessary to achieve solutions in some of the conditions.

Reviewer 2 comments with replies under:

The reviewer raises several minor points.

1) **We have used Greek characters (L116) and Roman nomenclature (L149), and this should be flagged more clearly.**

   Reply: Done – see lines 165 as an example. In addition, it is consistent throughout the paper as seen from the diff file.

2) **Fig. 3B left-hand panels would be clearer if the labels (e.g. (-1.2, 1.2) mV) were inside the associated box.**

   Reply: We found that it is hard to put them inside, so we increased the separation between plots.

3) **Fig 6. These are complex findings presented in a fairly sketchy manner. It would be interesting to see more raster style plots for the outputs of the bursts to see what variability there is within the family of 011 outputs (for instance), and whether this tells us anything useful?**

   Reply: Added to supplementary materials section 5.

4) **Related to the abbreviated presentation, it is not clear to me in Fig. 6c why the delays become shorter when the output spikes are produced later.**

Reply: This last point is quite an important one, as it reflects an error on our part, as we had reversed the sign in our output. The reviewer is quite right that the delay should become longer when output spikes are produced later, and indeed this is what we found, as corrected now and made clear in the updated figure.

Extra points:

1. The simulations in this work can take up to 3+ weeks (I didn't time it exactly) on four 3090 GPUs.
2. Some of the results were obtained on a smaller PC with two Titan GTX cards. A decision later came to unify all the results on the same workstation, so there are some changes, but the patterns of results are the exactly same. The changes are to Figure 2 (A, B and D), Figure 3 (A and B) and Figure 6 C.
3. On line 120, τ^{ap} was given the wrong value of 2 ms, now changed to the correct value of 4 ms.
4. Corrected some labels in the figures.
5. Code url: https://github.com/biocomplab/Neuro-morphology
6. The colour of the no solution is changed to "Tan" in Figures 2, 4, and 6.
